# A multi-site cross-sectional study on the burden of SARS-CoV-2 in healthcare workers in Madagascar

**DOI:** 10.1371/journal.pone.0309977

**Published:** 2024-10-24

**Authors:** Seth Kofi Abrokwa, Lantonirina Ravaoarisoa, Veronica Briesemeister, Radonirina Lazasoa Andrianasolo, Andry Maharo Andrianarivelo, Sophie Alice Müller, Zely Arivelo Randriamanantany, Andrea Bernasconi, Sabrina Weiss

**Affiliations:** 1 Center for International Health Protection, Robert Koch Institute, Berlin, Germany; 2 Laboratoire d’Analyses Médicales Malagasy, Antananarivo, Madagascar; 3 Institut National de Santé Publique et Communautaire–Madagascar, Antananarivo, Madagascar; 4 Faculté de Médecine, Université d’Antananarivo, Antananarivo, Madagascar; Universitas Syiah Kuala, INDONESIA

## Abstract

The prevalence of infections and risk factors that go along with them give insights into the burden of disease and effectiveness of infection prevention and control strategies. In this study we investigated the burden of severe acute respiratory syndrome coronavirus 2 (SARS-CoV-2) and associated epidemiological factors in three regions of Madagascar among healthcare workers. Between May and June 2021, we conducted a multi-site cross-sectional study among healthcare workers in eight University Hospital Centers, during the local second wave and before the coronavirus disease 2019 vaccination campaign in three regional capitals of Madagascar. We collected demographic information and relevant SARS-CoV-2 exposure history and tested for both immunoglobulin G antibodies to SARS-CoV-2 spike protein, using enzyme-linked immunosorbent assay and active SARS-CoV-2 infection using real-time reverse transcription-polymerase chain reaction. A total of 1006 healthcare workers enrolled in the study out of which 53.8% tested positive for either acute infection or SARS-CoV-2 antibodies. Approximately 50% of the participants reported receiving inadequate training on SARS-CoV-2 and associated infection prevention and control measures, inadequate supply of Personal Protective Equipment (PPE) and discomfort when using available PPE. Prevalence of acute infection was 3.4% without statistically significant variation in the different regions or health facilities as well as the different profession groups and units of work. Average seroprevalence of SARS-CoV-2 IgG antibodies was 52.0%, varying between 47.8% and 53.3% across the different regions. No significant difference was observed for region, gender, profession, and different risk groups. Predictive multivariable model showed significant association between seropositivity and healthcare facility and age (p<0.05). Our results revealed high infection rate of SARS-CoV-2 in HCWs in all three selected regions of Madagascar. The high disease burden identified in the study population might characterize the extent of high undocumented infection rates in HCWs in other regions of Madagascar.

## Introduction

The global emergence of the severe acute respiratory syndrome coronavirus 2 (SARS-CoV-2) has exposed healthcare workers (HCWs) to critical occupational risks resulting in higher infection rates and deaths in this population [1, 2]. Compared to the general population, HCWs are considered to be at a higher risk for SARS-CoV-2 infection due to their occupational exposure to infectious droplets and other potentially infectious materials [1, 3, 4]. Once infected, they can put other HCW, patients, members of their households and communities at risk. The World Health Organization (WHO) estimates that there are high levels of undocumented coronavirus disease 2019 (COVID-19) infection rates and high unmeasured levels of excess deaths attributable to COVID-19 infection in HCWs [2]. To inform and strengthen infection prevention and control (IPC) strategies, extensive documentation of infection rates and other health outcome indicators in HCWs is required. As the pandemic progresses, the health and well-being of HCWs remain a growing concern due to the excessive occupational risk of exposure [5, 6]. This concern is especially valid since HCWs not only face physical but also mental challenges [7]. Due to the limited size of health workforce, the lack of or inadequate personal protective equipment (PPE), and insufficient testing opportunities this concern is even higher in low- and middle-income countries (LMICs) such as Madagascar [8, 9].

Madagascar is the world’s second-largest island nation located off the coast of East Africa with over 25 million population [8]. In March 2020, the first two SARS-CoV-2 infections were identified in Antananarivo, the country’s capital [8]. By September 2020, active cases of SARS-CoV-2 infections were found in all 22 regions of the country [8, 10]. The country experienced a significant increase in cases, and are ranked 23^rd^ as of 15^th^ May 2022 among other African nations in terms of cumulative incidence and case fatality rates, and number of critically ill individuals [11, 12].

Reports from Madagascar have revealed inadequate testing capacities and low testing rates countrywide [8, 13], which leads to underestimation of the burden of SARS-CoV-2 infection in the general population. The under-detection of circulating infections in the general population poses a significant threat to healthcare institutions and HCWs. This study investigated the burden of SARS-CoV-2 infections on HCWs by analysing epidemiological factors such as region, healthcare facility, profession, unit of work, and history of contact with laboratory confirmed or suspected COVID-19 patients. The results of this study were used to inform methodological strategies in a larger study on burden of SARS-CoV-2 infection and vaccine effectiveness in HCWs currently being carried out by the Center for International Health Protection at the Robert Koch Institute (RKI) and four partner countries namely Madagascar, Nigeria, Ivory Coast, and the Democratic Republic of Congo (https://ghpp.de/en/projects/bchw/?L=0).

## Materials and methods

### Study design and study setting

This study was initiated through a cooperation between the RKI, which lies within the portfolio of the German Federal Ministry of Health, and the Laboratoire d’Analyses Médicales Malagasy (LA2M), operating under the Ministry of Public Health in Madagascar. We conducted a cross-sectional study in HCWs between May and June 2021 in three regional capitals: Analamanga in the center of the country, Boeny in the western part and Atsinanana in the eastern part. These three regions are among six regions in Madagascar with University Hospital Centers (CHU) and recorded most cases of SARS-CoV-2 during the two local waves of the pandemic that affected the country. We collected data during the local second wave and before the launching of the COVID-19 vaccination campaign. The study was designed following the “Strengthening the Reporting of Observational Studies in Epidemiology (STROBE)” Statement [14]. Ethics approval was obtained from the ethics board of the Charité - Universitätsmedizin Berlin (EA1/033/21) and the Comité d’Ethique de la Recherche Biomédicale de Madagascar. Written informed consent was taken from all participants. All participants were informed regarding the need of publishing the results with anonymized participant information.

### Inclusivity in global research

Additional information regarding the ethical, cultural, and scientific considerations specific to inclusivity in global research is included in the Supporting Information (S1 Checklist).

### Study population and sampling

We conducted the study in HCWs from all university hospital centers that have been specifically dedicated to the management of COVID-19 cases within the selected regions. This included all university hospitals from Antsinanana (CHU Analakininina and CHU Morafeno) and Boeny (CHU Androva and CHU Mahavoky Atsimo), and a random selection of four out of 12 from Analamanga. (CHU Joseph Ravoahangy Andrianavalona (JRA), CHU Joseph Raseta Befelatanana (JRB), CHU Gynéco Obstétrique Befelatanana (GOB) and CHU Anosiala). Together, these hospitals serve a total population size of 1,869,864. Data collection took place between May 10–21, 2021. The study population consisted of HCWs per definition by WHO, i.e. medical and nursing staff/students, midwives, allied HCWs, auxiliary HCWs such as cleaning and laundry personnel, social workers, admission/reception clerks, patient porters, catering staff and administration staff [15]. All HCWs 18 years and above, willing to provide written informed consent to both taking the survey and providing a blood sample and nasopharyngeal swab were eligible to participate in the study. S1 Table provides an overview of the total population the different hospitals serve, bed capacities, and number of COVID-19 cases reported in the facility as of May 2021. HCWs were selected from each hospital by simple random selection, proportional to size of the workforce, from the list of all HCWs who agreed to be enrolled into the study. To calculate the sample size, an expected seroprevalence of 10% among HCWs was taken with a marginal sampling error of ±2% and a confidence level of 95%. A 10% drop-out or non-response rate was assumed to calculate a minimum sample size of 862 participants. The expected seroprevalence was based on a literature search and accounting for an expected rise in infection.

### Survey

An electronic survey was set up on the KoBoCollect survey platform (Kobo Inc., Cambridge, MA, USA) [16]. The survey was administered by trained interviewers. Relevant information was collected using a modified version of the questionnaire from the WHO Protocol for assessment of potential risk factors for COVID-19 among health workers in a healthcare setting [17]. The survey included demographic characteristics of HCWs, exposure to and clinical history of COVID-19, usage of PPE, and other risk factors such as comorbidities and medication history, as well as questions assessing the perception of HCWs towards SARS-CoV-2 IPC measure. Comorbidities included pregnancy, diabetes, cancer, HIV infection/ other immunodeficiencies, chronic pulmonary disease, asthma, hypertension, and cardiac diseases. The modified survey was translated from English to French and piloted in Madagascar among health professionals not included in the study.

### Collection and handling of biological samples

Nasopharyngeal swabs and a 10 mL blood sample were collected and processed by qualified local staff of LA2M, Madagascar. Preparation and processing of samples were conducted in an appropriately maintained and certified biosafety cabinet following the proper Standard Operating Procedures (SOPs) to allow sample manipulation while minimizing the risk of release and dissemination of virus containing aerosols, droplets, and materials before sample inactivation. In addition, all virus-related work was performed by personnel trained in the relevant technical and safety procedures. Standard international guidelines for laboratory biosafety were always followed along with wearing the appropriate PPE, i.e., scrub suits, shoe covers, disposable gloves, eye protection and a face mask.

### Laboratory testing

Acute infections with SARS-CoV-2 were tested using specimens collected by nasopharyngeal swab. Nucleic acid extraction was performed using the QIAamp Viral RNA Mini Kit (Qiagen, Germany) and tested for SARS-CoV-2 using a commercially available real-time reverse transcription-polymerase chain reaction (RT-PCR) assay targeting the E-Gene (Sarbecovirus SARS-CoV-2; TIB MolBiol, Berlin, Germany), following the manufacturer’s instructions. Positive tested samples were confirmed using the SARS-CoV-2 RdRP assay by the same manufacturer. Participants that tested positive for active infection with SARS-CoV-2 were informed immediately and requested to follow the national COVID-19 directives and protocols.

An enzyme-linked immunosorbent assay (ELISA) was used to test for anti‐SARS‐CoV‐2 IgG antibodies in serum samples. The Euroimmun IgG ELISA (Euroimmun, Lübeck, Germany) was used as a first-line screening test. Briefly, this semi-quantitative assay uses a plate coated with the S1 domain of the spike protein to detect IgG and was performed using the instructions provided by the manufacturer. Reactive samples were subsequently confirmed with the Wantai SARS-CoV-2 Ab ELISA (Beijing Wantai Biological Pharmacy Enterprise, Beijing, China). This is a qualitative assay for the detection of total antibodies, including IgG and IgM. A sample was considered positive only if reactive in both tests.

### Data handling and statistical analysis

We merged serological and RT-qPCR data with survey data through the participants’ identification numbers and extracted anonymized data as Microsoft Excel file for analysis. All statistical analysis was conducted using R (version R-4.1.2).

HCWs were categorized into two groups depending on the presumed risk of infection (high and low) based on the history of contact with COVID-19 patients in the health facility according to the WHO definition [5]. High risk groups were defined as those with contact with a PCR confirmed or suspected COVID-19 patient within one meter and for a prolonged period of more than 15 minutes. For the variable profession, all professions held by fewer than 10 HCWs were aggregated in two sub-categories: “clinical auxiliary HCWs” consisting of medical/dental assistants, phlebotomists, dispensary staff, morgue staff, physiotherapists, and nutritionists/dieticians, and “non-clinical auxiliary HCWs” comprising security staff, maintenance personnel, catering, cleaners, social assistants, patient carriers, admission clerks, and paramedics. We reclassified the unit of work into three main categories, namely COVID-unit only, clinical non-COVID unit (i.e., hygiene unit, emergency/high dependency unit (HDU)/ intensive care unit (ICU), laboratory unit, maternity unit, medical unit, surgical unit, pharmacy unit, and radiology unit), and administrative unit. Because a Likelihood Ratio (LR) test produced no evidence for a linear trend in log odds through, age was grouped in 5 age groups. Frequencies are provided for the analysis of the perception of HCWs towards SARS-CoV-2 IPC measures.

The seroprevalence of SARS-CoV-2 antibodies and prevalence of SARS-CoV-2 active infection were computed and reported as counts and proportions with 95% confidence intervals (95% CI). The burden of SARS-CoV-2 among HCWs was estimated as the proportion of HCWs who tested positive for acute infection by PCR or past infection by SARS-CoV-2 serology. We focused on seropositivity as outcome for further risk factor analysis to facilitate the comparison with other studies and available population data from Madagascar. Logistic regression was used to obtain crude odds ratios (cOR), 95% confidence intervals (95% CI), and *p* values, to evaluate associations between seropositivity and the explanatory variables including region, healthcare facility, age, gender, profession, work unit, and risk group. All bivariable associations with *p* values ≤ 0.1 were selected into the initial saturated logistic regression model for a predictive multivariable analysis to identify predictors and to determine the strengths of association for these variables based on the derived adjusted odds ratios (aOR). The reference group was HCWs in CHU Morafeno who were in the age category 18–29, and work in the administrative unit. A backward stepwise selection process based on the likelihood ratio test was adopted to compare statistical models considering the different covariates. All performed tests were two-tailed and statistical significance was set at p < 0.05.

## Results

### Sociodemographic characteristics of participants

A total of 1006 HCWs responded to the survey and provided nasopharyngeal swabs as well as blood samples for laboratory testing. The distribution of HCWs in the different regions and healthcare facilities is presented in Table 1. The median age of participants was 38.1 (IQR, 29.6–46.3) with a majority in the age category of 30–39 (Table 2). Females made up 63.9% of the HCWs. Among the different profession groups, 22.1% were medical doctors, 14.9% nurses, 12.0% midwives, 2.0% laboratory staff, 3.1% radiology staff, and 14.0% administrative staff. Clinical auxiliary staff made up 4.9%, while non-clinical auxiliary HCW made up 27.1% of all participants. Cumulatively, 12.1% of the participants worked in COVID-19 units.

**Table 1:** Distribution of HCWs in the three regions and selected health facilities with corresponding seroprevalence of SARS-CoV-2 antibodies and prevalence of acute infections (PCR positive).

Demographic characteristics	HCWs n (%)	HCWs in COVID Units # (%)	SARS-CoV-2 IgG pos n (%)	Crude OR (95% CI)	p-value	PCR pos n (%)	p-value
**Overall**	1006 (100)	122 (12.1)	524 (52.1)			34 (3.4)	
**Region**							
Boeny	157 (15.6)	17 (10.8)	74 (47.8)		ref	4 (3.2)	
Atsinanana	160 (15.9)	20 (12.5)	82 (51.2)	1.15 (0.74–1.79)	0.54	8 (5.0)	
Analamanga	689 (68.5)	85 (12.3)	367 (53.3)	1.25(0.88–1.77)	0.21	22 (2.5)	
					0.45*		0.43**
**Health facility**							
CHU Morafeno	59 (5.9)	3 (5.1)	23 (39.0)		ref	4 (6.8)	
CHU Androva	105 (10.4)	14 (13.3)	49 (46.7)	1.37 (0.72–2.64)	0.34	2 (1.9)	
CHU MahavokyAtsimo	52 (5.2)	3 (5.8)	25 (50.0)	1.57 (0.74–3.35)	0.24	2 (3.8)	
CHU JRA	260 (25.8)	22 (8.5)	128 (49.2)	1.52 (0.86–2.73)	0.16	7 (2.7)	
CHU Anosiala	76 (7.6)	14 (18.4)	38 (50.0)	1.56 (0.79–3.14)	0.20	5 (6.6)	
CHU GOB	104 (10.3)	10 (0.6)	56 (53.8)	1.83 (0.96–3.53)	0.07	4 (3.8)	
CHU JRB	249 (24.8)	39 (15.7)	145 (58.2)	2.18 (1.23–3.94)	<0.01	6 (2.4)	
CHU Analakininina	101 (10.0)	17 (16.8)	59 (58.4)	2.19 (1.15–4.28)	0.02	4 (3.9)	
					0.10*		0.49**

HCW: Healthcare workers, CI: Confidence interval, PCR: Polymerase chain reaction, ref: reference category, pos: positive.

Overall p-value for each bivariable analysis, significance at p < 0.05 by chi-square test** or Fischer exact test are indicated in bold

**Table 2:** Baseline characteristics of study participants with seroprevalence of SARS-CoV-2 antibodies and prevalence of acute infections (PCR positive).

Demographic characteristics	HCWs # (%)	SARS-CoV-2 IgG pos # (%)	Crude OR (95% CI)	p-value	PCR pos # (%)	p-value
**Gender**						
Male	363 (36.1)	187 (51.5)		ref	11 (3.0)	
Female	643 (63.9)	336 (52.4)	1.04 (0.80–1.34)		23 (3.6)	
				**0.78** ^*^		0.84**
**Age in categories**						
18–29	266 (26.4)	140 (51.8)		ref	6 (2.6)	
30–39	303 (30.1)	143 (47.8)	0.85 (0.61–1.18)	0.34	10 (3.3)	
40–49	266 (26.4)	148 (55.6)	1.16 (0.83–1.64)	0.38	13 (4.8)	
50–59	151 (15.0)	85 (56.9)	1.23 (0.82–1.84)	0.32	3 (1.8)	
60+	20 (2.0)	7 (35.0)	0.50 (0.18–1.26)	0.15	2 (5.3)	
				**0.10** ^*^		0.42**
**Profession**						
Administrative staff	140 (14.0)	68 (48.6)		ref	4 (2.9)	
Clinical auxiliary HCW	49 (4.9)	26 (53.1)	1.20 (0.62–2.31)	0.59	3 (6.1)	
Non-clinical auxiliary HCW	273 (27.1)	138 (50.7)	1.09 (0.73–1.64)	0.68	7 (2.6)	
Laboratory staff	20 (2.0)	14 (70.0)	2.47 (0.93–7.32)	0.08	1 (5.0)	
Medical doctor	221 (22.1)	111 (50.2)	1.07 (0.70–1.63)	0.76	7 (3.2)	
Midwife	120 (12.0)	69 (57.5)	1.43 (0.88–2.35)	0.15	2 (1.7)	
Nurses	150 (14.9)	83 (55.7)	1.33 (0.84–2.12)	0.23	7 (4.7)	
Radiology staff	33 (3.1)	14 (45.2)	0.87 (0.39–1.90)	0.73	3 (9.7)	
				**0.48** ^*^		0.37**
**Unit of work**						
Administrative unit	82 (9.0)	38 (46.3)		ref	4 (4.9)	
COVID unit	122 (12.1)	71 (58.2)	1.61 (0.92–2.84)	0.51	7 (5.3)	
Hygiene unit	29 (3.2)	17 (58.6)	1.64 (0.7–3.94)	0.09	1 (3.4)	
Emergency/HDU/ICU unit	110 (12.0)	61 (55.5)	1.44 (0.81–2.57)	0.26	3 (37)	
Laboratory unit	39 (4.3)	24 (61.5)	1.85 (0.86–4.09)	0.21	2 (51.3)	
Maternity unit	84 (9.2)	50 (59.5)	1.70 (0.92–3.17)	0.12	5 (6.0)	
Medical Ward unit	277 (30.2)	134 (48.4)	1.09 (0.66–1.78)	0.09	5 (1.8)	
Pharmacy unit	53 (5.8)	33 (62.3)	1.91 (0.95–3.91)	0.75	1 (1.9)	
Radiology unit	60 (6.6)	26 (46.7)	1.01 (0.52–1.98)	0.09	2 (3.3)	
Surgical ward unit	60 (6.6)	20 (33.3)	0.58 (0.29–1.15)	0.12	4 (6.6)	
				**0.02** ^*^		
**Work unit, regrouped**						
Nonclinical	353 (35.1)	188 (53.2)		ref		
Clinical	531 (52.8)	365 (49.9)	0.87 (0.67–1.14)	0.33		
COVID unit	122 (12.1)	71 (58.2)	1.22 (0.81–1.86)	0.35		
				**0.22** ^*^		
**Risk group**						
Low risk	592 (58.8)	299 (50,5)		ref	16 (2.7)	
High risk	414 (41.2)	225 (54,2)	0.86 (0.67–1.1)	**0.23** ^*^	13 (4.3)	
						0.61**
**Comorbidity**						
No	667 (66.4)	346 (51.8)		ref	13 (3.1)	
Yes	338 (33.6)	178 (52.7)	1.04 (0.80–1.35)	**0.80**	21 (3.8)	0.88**
**Obesity**						
No	890 (90.6)	450 (50.6)		ref	29 (3.3)	
Yes	92 (9.4)	60 (65.2)	1.83 (1.18–2.89)	**0.008** ^*^	5 (5.4)	0.36**
**COVID Symptoms**						
No	659 (65.6)	-			18 (2.7)	
Yes	346 (34.4)	-			16 (4.6)	0.16**

HDU: High dependency unit, ICU: Intensive care unit, PCR: Polymerase chain reaction, ref: reference category, pos: positive.

Overall p-value for each bivariable analysis, significance at p < 0.05 by chi-square test^*^ or Fisher exact test^**^ are indicated in bold

^$^Obesity measured as body mass index (BMI) > 30kg/m^2^

### Risk perception and use of protective equipment

54.7% of the participants reported to have received training on SARS-CoV-2 and its associated infection prevention measures since the beginning of the pandemic (S2 Table). In total, 54.8% of the participants noted that SARS-CoV-2 infection prevention training programs were inadequate. Regarding workload, 61.8% and 12.1% strongly agreed and agreed to an increase in their workload, respectively, during the epidemic. 29.9% and 31.7% of the participants strongly disagreed and disagreed, respectively, that there has been adequate supply of PPE since the beginning of the pandemic. Furthermore 14.6% and 51.1% reported that available PPEs were very uncomfortable and uncomfortable to use, respectively. The majority (46.6%) responded that they were dissatisfied with the PPEs available. A total 49.0% and 29.0% reported having moderate and low level of protection, against infection respectively. 4.27% of the participants reported having very low level of protection. 58.6% (n = 589) of the HCWs reported high risk of infection with SARS-CoV-2, while 34.5% (n = 347) rated their risk as moderate.

### Burden of SARS-CoV-2 among study participants

Overall, there was a high SARS-CoV-2 burden of 53.8% (n = 541) when previous infection or current infections were taking into consideration in our study population. This high disease burden varied across the different regions, health facilities, gender, profession, and unit of work.

### SARS-CoV-2 prevalence

**Fig 1:**
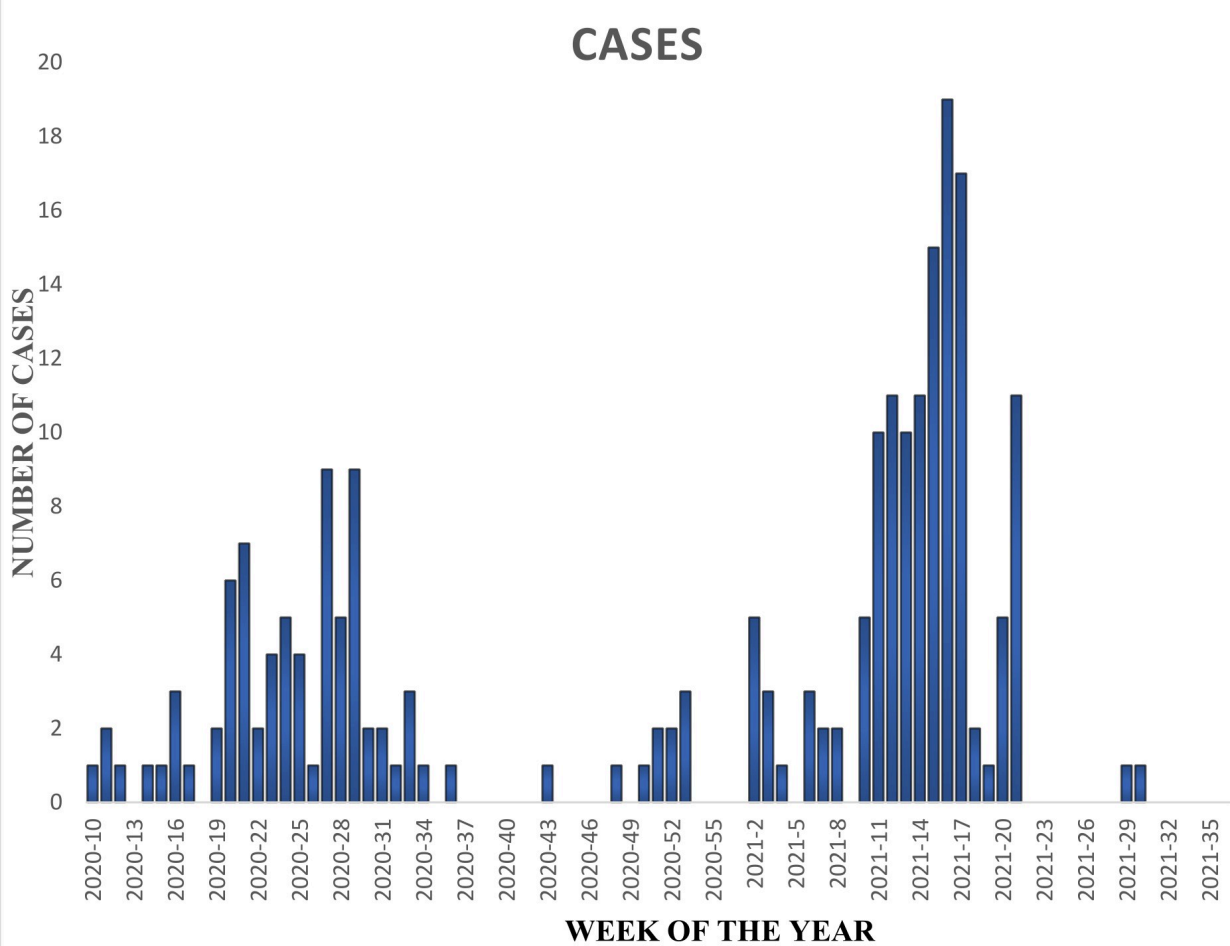
Epidemic curve. Self-reported dates of symptom onset from study participants testing PCR positive for SARS-CoV-2 since the beginning of the Pandemic in the eight University hospital centers in Madagascar.

Overall, 3.4% HCWs tested positive for acute SARS-CoV-2 infection by PCR (Table 1). Across the regions, the prevalence of acute infection ranged from 2.5% in Analamanga to 5.0% in Atsinanana (p = 0.43). Amongst the different health facilities, CHU Morafeno recorded the highest (6.8%) and CHU Androva recorded the lowest prevalence (1.9%, p = 0.49). There was no significant difference between gender (females 3.6%, males 3.0%, p = 0.84) (Table 2). Highest rate of acute infection was recorded among the age category 40–49 (4.8%). The proportion of confirmed current infection was higher in those who reported COVID symptoms (4.6%) than in those who did not report such symptoms (2.7%, p = 0.16). Further analysis of the symptoms revealed significant association between prevalence of acute SARS-CoV-2 infection and nausea (p = 0.02) and arthralgia (p<0.001) (S3 Table). 21.8% (n = 219) of the study population reported testing PCR positive for SARS-CoV-2 infection since the beginning of the pandemic as illustrated by the epidemic curve (Fig 1).

### Seroprevalence of SARS-CoV-2 among HCWs

In total, 523 (52.1% [95%CI, 48.9%– 55.2%]) individuals tested positive for SARS-CoV-2 IgG antibodies. Table 1 shows seroprevalence of SARS-CoV-2 IgG antibodies in HCWs in the different regions and health facilities. Seroprevalence varied in the three study regions between 47.8% and 53.3%. Across the eight different healthcare facilities, seroprevalence ranged from 39.0% to 58.4% (p = 0.10). Bivariate analysis showed that HCWs in CHUs Analakininina (OR, 2.19; [95%CI, 1.15–4.28] and JRB (OR, 2.18; [95%CI, 1.23–3.94]) have significantly higher odds of being infected with SARS-CoV-2 as compared to those in other regions. When separating by profession, the highest seroprevalences were detected among laboratory staff (70.0%) followed by midwives (57.5%) and nurses (55.7%). The lowest seroprevalence was found in radiology staff (45.2%) and administrative staff (48.6%). There was no significant association between seroprevalence of SARS-CoV-2 antibodies and the different professional categories (p = 0.47). Seroprevalence varied in HCWs in the different units of work. 61.5% of HCWs in the laboratory unit tested positive for SARS-CoV-2 IgG antibodies being the highest rate, followed by 62.3% in the pharmacy unit, 59.5% maternity unit, 58.6% in the hygiene unit and 58.2% in the COVID unit.

HCWs in the surgical and medical units had the lowest seroprevalence of 33.3% and 48.4%, respectively. Bivariate analysis revealed a statistically significant association between seroprevalence and the unit of work (p = 0.02). Following the recategorization of the working units into three separate groups, the COVID-19 unit had the highest seroprevalence of 58.2% (n = 71), but no significant association between seropositivity and reclassified groups was found as shown in Table 2. Among the high-risk category, 54.2% were seropositive for SARS-CoV-2 antibodies, while this proportion amounted to 50.5% among 592 HCWs with low-risk contacts only (p = 0.23). Among 338 participants who reported at least one comorbidity, 52.7% tested positive for SARS-CoV-2 antibodies, whereas 51.8% among those without comorbidity tested positive for SARS-CoV-2 antibodies. The analysis found no statistically significant association between seroprevalence and the perception of participants towards SARS-CoV-2 IPC measures.

The predictive multivariable model included the variables healthcare facility, and age group, and working unit, as shown in Table 3. Compared to the control group, statistically significant higher odds of seropositivity were found in HCWs in CHU Analakininina (aOR, 3.02; [95%CI, 1.47–6.36], CHU JRB (aOR, 2.67; [1.41–5.21]), and CHU GOB (aOR, 2.43; [1.19–5.05]). Borderline significant odds of seroprevalence of SARS-CoV-2 antibodies were found in HCWs in CHU JRA (aOR, 2.08; [1.10–4.02]) compared to the control group. Statistically significant lower odds of seroprevalence of SARS-CoV-2 antibodies were found in HCWs older than 60 years old (aOR, 0.35; [0.12–0.87])

**Table 3:** Crude odds ratio and adjusted odds ratios and 95% confidence intervals from multivariable logistic regression analysis for seropositivity among HCWs adjusting for health facility, age group and place of work.

Characteristics	cOR	95% CI	p-value	aOR	95% CI	p-value
**Health care facility**						
CHU Morafeno						ref
CHU Androva	1.37	0.72–2.64	0.34	1.73	0.85–3.56	0.195
CHU MahavokyAtsimo	1.57	0.74–3.35	0.24	2–02	0.90–4.61	0.100
CHU JRA	1.52	0.86–2.73	0.16	2.08	1.10–4.02	0.051
CHU Anosiala	1.56	0.79–3.14	0.20	2.38	1.12–5.15	0.063
CHUGOB	1.83	0.96–3.53	0.07	2.43	1.19–5.05	0.022
CHU JRB	2.18	1.23–3.94	<0.01	2.67	1.41–5.21	0.006
CHU Analakininina	2.19	1.15–4.28	0.02	3.02	1.47–6.36	0.005
**Age group**						
30–40	0.85	0.61–1.18	0.34	0.92	0.64–1.33	0.770
40–50	1.16	0.83–1.64	0.38	1.14	0.78–1.68	0.865
50–60	1.23	0.82–1.84	0.32	1.33	0.84–2.13	0.200
60+	0.50	0.18–1.26	0.15	0.35	0.12–0.87	0.026
**Unit of work**						
COVID unit	1.61	0.92–2.84	0.10	1.43	0.89–2.91	0.285
Hygiene unit	1.64	0.7–3.94	0.26	1.38	0.69–4.01	0.435
Emergency/HDU/ICU	1.44	0.81–2.57	0.21	1.47	0.84–2.72	0.388
Laboratory	1.85	0.86–4.09	0.12	1.81	0.87–4.30	0.160
Maternity	1.70	0.92–3.17	0.09	1.60	0.93–1.35	0.405
Medical Ward	1.09	0.66–1.78	0.75	1.03	0.71–1.65	0.938
Pharmacy	1.91	0.92–3.8	0.07	2.23	1.10–4.75	0.080
Radiology	1.01	0.52–1.98	0.97	0.99	0.54–2.14	0.854
Surgical ward	0.58	0.29–1.15	0.12	0.57	0.29–1.21	0.086

p-value significance at <0.05 by Wald test, cOR: crude odds ratio, aOR: adjusted odds ratio, CI: Confidence interval, ref: reference category.

## Discussion

During the first and second local wave of the COVID-19 pandemic the government in Madagascar put in place specific strategies to contain the spread of the virus, including several non-pharmacological interventions, and improving testing capacities. While it is argued that the total number of cases of COVID-19 may be underestimated or under-reported due to limited testing capacity [13, 18], the extent of the burden of the ongoing pandemic in HCWs in Madagascar is unclear. Here, we report the burden of SARS-CoV-2 infection in a sample of HCWs from eight tertiary hospitals dedicated to the management of COVID-19 cases, during the second local wave of the COVID-19 pandemic and before the launching of the COVID-19 vaccination campaign in three of the most affected regions: Analamanga, Atsinanana, and Boeny. Our study revealed a high burden of disease in HCWs of 53.8%. As COVID-19 vaccination was not available in Madagascar at the time of data collection, we are using the presence of SARS-CoV-2 IgG antibodies as a proxy for disease burden to allow for data comparison. Our study shows an average seroprevalence of 52% with statistically significant variations (seroprevalence ranging between 39.0% and 58.4%) depending on the healthcare facility, age category, and obesity as a comorbid condition. The observed seroprevalence of SARS-CoV-2 in the HCWs was considerably higher compared to global meta-analyses estimates and other similar studies in low-income settings (up to 26.8%) [19–22]. However, high seroprevalence rates in HCWs in Antananarivo, Madagascar (36.6%) [23] and other African regions (up to 45.1%) [24], South America (up to 71%) [25, 26] and other high income countries including Spain and the UK (up to 31%) [27, 28] have been reported. We found significant differences in the distribution of seroprevalence between the healthcare facilities; highest seroprevalence rates were observed among HCWs in three out of four health facilities in the most populated region, Analamanga, (CHU JRB, CHU JRA and CHU GOB).

Specific factors determining high seroprevalence rates vary and data from our study do not allow for direct comparison within the general population. However, estimates of SARS-CoV-2 seroprevalence in blood donors from regional blood transfusion centers of these three regions show equally high numbers: Analamanga (blood donors 63.1% vs 53.3% in HCW), Atsinanana (blood donors 43.4% vs 51.2% in HCW), and Boeny (blood donors 35.5% vs 47.8% in HCW) [29, 30]. Using the blood donors as a proxy for the general population, we could infer from these findings that there is high circulation of SARS-CoV-2 in the general population which may account for the high seroprevalence in HCWs. In addition, our analysis found no difference in seroprevalence in HCWs after stratifying by region, gender, and profession. Further stratification by HCWs working in COVID-19 units compared to HCWs working in non-COVID-19 units as well as risk categorization did not yield any significant difference in seroprevalence rates. These findings are also suggestive of high community transmission rather than transmission in the health facilities. A similar conclusion was reached by a study which investigated seroprevalence of SARS-CoV-2 in HCWs in Kenya [31]. Additional studies have identified similar trends of high transmission from the general population to HCWs [32, 33].

In our analysis, we identified other important details which could contribute to the high seroprevalence in HCWs aside from the community transmission theory. HCWs reported of inadequate supply of PPE, which resonates with the global shortage of PPE due to increased demand which led to depleted stockpiles worldwide. Most LMIC have not had access to adequate supply of PPEs due to the limited resources and infrastructural weakness of their health systems [34]. Limited supply of PPE could account for frontline HCWs being ill-equipped to provide care for COVID-19 patients. Similarly, the majority of HCWs in our study noted the difficulties in using the available PPEs, as they described them as “uncomfortable to use*”*. The limitation of our study design made it impossible to ascertain further the factors associated with “uncomfortable to use PPEs*”*. In some studies, however, gender inequity in the provision of PPE was identified to play a role in high seroprevalence among women due to ill-fitting PPE, which are predominantly designed based on male body shape [35, 36]. Given that the PPE available to our study population are not produced locally, it is plausible to reason that the variation in the body habitus based on which the PPE were produced can lead to some discomfort in donning or doffing. This also holds true for most LMICs and even other industrial countries where there are structural inequities in the production of PPE as mentioned above. The end effect will be loosely fitting PPEs not appropriate for infection prevention, or extremely tight PPE which can lead to pressure sores and therefore unpleasant to don [37]. Consequently, ill-fitting PPE due to poor donning (including masks and googles that fail to seal or inadequately sized gloves and gowns), rationing of PPE and prolonged use can put HCWs at an increased risk of infection. The current pandemic has exposed gaps in global health systems’ response and many countries are struggling to deal with its socio-economic consequences. Far more so, it has revealed structural inequities that exist in terms of testing capacities, treatment opportunities, vaccination, and production and provision of PPE [38–40]. The pandemic has uncovered the need for global health actors to collaborate towards improving and strengthen health systems response in IPC initiatives especially by considering the structural inequities (including gender and race) that exists in the production and provision of PPE [4, 41].

We further assessed the role of gender, age, profession, and work unit of HCWs. High seropositivity did not differ by gender and profession. Those working in the pharmacy unit were observed to have higher seropositivity compared to administration staff. Given the nature of their work and how the healthcare facilities are set-up, HCWs in pharmacies have more frequent contact with patients or their families because of the tasks they perform (delivery of medicines). This could account for the high seroprevalence in this group if we consider that there is high circulation of the virus in the general population. Our findings further suggest that being older than 60 years is associated with lower seroprevalence among the study population. Compared to the young, HCWs above 60 years of age were less represented in the study and interpretation of the results should be done with caution. Other studies have also reported similar findings of low seroprevalence in similar age groups of HCWs [42–44].

A particular strength of our study is the collection of data at the climax of the second wave and before vaccination was initiated. The evidence provides understanding into the extent of disease burden in HCWs and highlights the possibility of high disease burden in the community. It further reveals to some extent the gaps in the infection and prevention strategies such limited supply PPE, which are deemed inadequate and uncomfortable for work therefore putting HCWs at high occupational risk. Another strength of our study is using Wantai ELISA as confirmation to minimize testing error.

We acknowledge that our study has several limitations. The study was limited to the tertiary healthcare facilities dedicated to managing COVID-19 cases therefore limiting the generalizability of the results to HCWs in other workplaces, especially healthcare facilities not designated to manage COVID-19 cases including the primary care setting. Given that the Ministry of Health in Madagascar adopts the syndromic approach for COVID-19 case management during epidemic waves, undetected asymptomatic case poses significant risk to HCWs especially in the low level of care. Secondly, all demographic, clinical information, history of exposure with COVID-19 cases, and use of PPE were self-reported and thus susceptible to recall and reporting bias. Thirdly, the aggregation of the profession group in our analysis is likely to obscure the true rates on infection in some sub-categories of HCWs.

In summary, our results revealed a high burden of SARS-CoV-2 infection in HCWs in all three selected regions of Madagascar, which reaffirm the WHO`s claims of under-documentation of SARS-CoV-2 infection rates in HCWs. The high burden of disease identified in the study population characterizes the extend of undocumented infection rates in general health work force in Madagascar. The findings of our study present an opportunity for policy makers and other health stakeholders in Madagascar to review current IPC strategies. It would be essential to adopt locally adaptable strategies to create and increase awareness on IPC measures not only among HCWs, but also in the general population to control community spread. Developing appropriate solutions for providing suitable PPE to HCWs is crucial to allow for efficient patient care while minimizing the risk of exposure to infection. This will also involve increasing and improving testing capacities for early detection and management among HCWs. These interventions together with access to vaccination ensures a safe working environment for HCWs who are integral to the response to the pandemic.

## Supporting information

S1 Checklist Inclusivity in global research. (DOCX)

S1 Table Overview of the eight University Hospital Center in the three regions of Madagascar. (DOCX)

S2 Table Health care workers perception of SARS-CoV-2 and related infection prevention measures with corresponding seroprevalence of SARS-CoV-2 IgG antibodies and prevalence of acute SARS-CoV-2 infection during the pandemic. (DOCX)

S3 Table Overview of symptoms reported by health care workers within 4 weeks before PCR test for acute infection. (DOCX)
