# Supplementary material for: A multi-site cross-sectional study on the burden of SARS-CoV-2 in healthcare workers in Madagascar
Source: PLoS One. 2024 Oct 24;19(10):e0309977. doi: 10.1371/journal.pone.0309977 (PMC11500896; doi:10.1371/journal.pone.0309977)
Supplement: S1 Table — (DOCX) [file pone.0309977.s002.docx]

**S2 Table. Overview of the eight University Hospital Center in the three regions of Madagascar.**

| **Regions** | **University Hospital Center (CHU)** | **Bed capacities** | **Total population served** | **Number of Covid-19 patients recorded between the beginning of the pandemic March 2020 and February 2022** |
| --- | --- | --- | --- | --- |
| Atsinanana | CHU Androva | 317 | 246,022 | 44 |
|  | CHU Mahavoky Atsimo | 163 |  | 109 |
| Boeny | CHU Analakininina | 417 | 325,857 | 209 |
|  | CHU Morafeno | 80 |  | 205 |
| Analamanga | CHU Joseph Ravoahangy Andrianavalona (JRA) | 650 | 1,297,985 | 272 |
|  | CHU Joseph Raseta Befelatanana (JRB) | 368 |  | 788 |
|  | CHU Anosiala | 100 |  | 434 |
|  | CHU Gynéco Obstétrique Befelatanana (GOB) | 100 |  | 32 |
