# Supplementary material for: A multi-site cross-sectional study on the burden of SARS-CoV-2 in healthcare workers in Madagascar
Source: PLoS One. 2024 Oct 24;19(10):e0309977. doi: 10.1371/journal.pone.0309977 (PMC11500896; doi:10.1371/journal.pone.0309977)
Supplement: S2 Table — (DOCX) [file pone.0309977.s003.docx]

**S3 Table. Health care workers perception of SARS-CoV-2 and related infection prevention measures with corresponding seroprevalence of SARS-CoV-2 IgG antibodies and prevalence of acute SARS-CoV-2 infection during the pandemic.** *Chi-square test or Fisher exact test was used to produce p-values where appropriate, ** N<1006 because statement was not applicable to all interviewees.

| **CHARACTERISTICS**  **N=1006, unless stated otherwise** | **Total number of health workers n (%)** | **SARS-CoV-2 IgG positive n (%)** | **p-value*** | **PCR positive n (%)** | **p-value*** |
| --- | --- | --- | --- | --- | --- |
| **IPC Training** |  |  |  |  |  |
| Less than 30 days ago | 74 (7.4) | 36 (48.6) |  | 3 (4.1) |  |
| 1 to 5 months ago | 127 (12.6) | 64 (50.4) |  | 2 (1.6) |  |
| 6 to 12 months ago | 133 (13.2) | 66 (49.6) |  | 5 (3.8) |  |
| More than a year ago | 216 (21.5) | 107 (49.5) |  | 10 (4.6) |  |
| Don’t remember | 174 (17.3) | 100 (57.5) |  | 7 (4.0) |  |
| No training | 282 (28.0) | 151 (53.5) | 0.68 | 7 (2.5) | 0.62 |
| **Adequate IPC training** |  |  |  |  |  |
| Strongly Agree | 115 (11.4) | 58 (50.4) |  | 2 (1.7) |  |
| Agree | 121 (12.0) | 61 (50.4) |  | 7 (5.8) |  |
| Disagree | 384 (38.2) | 197 (51.3) |  | 10 (2.6) |  |
| Strongly Disagree | 167 (16.6) | 84 (50.3) |  | 9 (5.4) |  |
| Not applicable | 219 (21.8) | 124 (56.6) | 0.67 | 6 (2.7) | 0.19 |
| **Increase in workload** |  |  |  |  |  |
| Strongly Agree | 622 (61.8) | 318 (51.1) |  | 20 (3.2) |  |
| Agree | 122 (12.1) | 57 (46.7) |  | 6 (4.9) |  |
| Disagree | 229 (22.8) | 131 (57.2) |  | 7 (3.1) |  |
| Strongly Disagree | 33 (3.3) | 18 (54.5) | 0.25 | 1 (3) | 0.80 |
| **Adequate supply of PPE** |  |  |  |  |  |
| Strongly Agree | 167 (16.6) | 83 (49.7) |  | 2 (1.2) |  |
| Agree | 219 (21.8) | 119 (54.3) |  | 7 (3.2) |  |
| Disagree | 319 (31.7) | 162 (50.8) |  | 11 (3.4) |  |
| Strongly Disagree | 301 (29.9) | 160 (53.2) | 0.76 | 14 (4.7) | 0.27 |
| **Comfort level of PPE (N=865)**** |  |  |  |  |  |
| Very comfortable | 72 (8.3) | 36 (50.0) |  | 2 (2.8) |  |
| Comfortable | 225 (26.0) | 113 (50.2) |  | 6 (2.7) |  |
| Uncomfortable | 442 (51.1) | 233 (52.7) |  | 12 (2.7) |  |
| Very uncomfortable | 126 (14.6) | 66 (52.4) | 0.924 | 10 (7.9) | 0.06 |
| **Satisfaction with quality of PPE** |  |  |  |  |  |
| Strongly Agree | 69 (6.9) | 32 (46.4) |  | 1 (1.4) |  |
| Agree | 423 (42.0) | 226 (53.4) |  | 13 (3.1) |  |
| Disagree | 469 (46.6) | 245 (52.2) |  | 19 (4.1) |  |
| Strongly Disagree | 45 (4.5) | 21 (46.7) | 0.628 | 1 (2.2) | 0.63 |
| **Confidence in IPC protocols** |  |  |  |  |  |
| High | 230 (22.9) | 115 (50) |  | 4 (1.7) |  |
| Moderate | 248 (24.7) | 124 (50) |  | 9 (3.6) |  |
| low | 366 (36.4) | 124 (50) |  | 18 (4.9) |  |
| Very low | 162 (16.1) | 90 (55.6) | 0.61 | 3 (1.9) | 0.12 |
| **Level of protection against SARS-CoV-2** |  |  |  |  |  |
| High | 178 (17.7) | 91 (51.1) |  | 4 (2.2) |  |
| Moderate | 493 (49.0) | 268 (54.4) |  | 18 (3.7) |  |
| Low | 292 (29.0) | 150 (51.4) |  | 10 (3.4) |  |
| Very low | 43 (4.3) | 15 (34.9) | 0.10 | 2 (4.7) | 0.80 |
| **Risk of infection with SARS-CoV-2** |  |  |  |  |  |
| High | 589 (58.5) | 330 (56) |  | 22 (3.7) |  |
| Moderate | 347 (34.5) | 162 (46.7) |  | 12 (3.5) |  |
| Low | 51 (5.1) | 24 (47.1) |  | 0 |  |
| Very low | 19 (1.9) | 8 (42.1) | 0.44 | 0 | 0.44 |
