# Supplementary material for: A multi-site cross-sectional study on the burden of SARS-CoV-2 in healthcare workers in Madagascar
Source: PLoS One. 2024 Oct 24;19(10):e0309977. doi: 10.1371/journal.pone.0309977 (PMC11500896; doi:10.1371/journal.pone.0309977)
Supplement: S3 Table — (DOCX) [file pone.0309977.s004.docx]

**S4 Table. Overview of symptoms reported by health care workers within 4 weeks before PCR test for acute infection.** Fisher exact test was used to produce p-values, PCR: Polymerase chain reaction.

| **Symptoms** | **Number of health workers reporting n (%)** | **PCR Positive n (%)** | **P value** |
| --- | --- | --- | --- |
| **Fever** |  |  |  |
| No | 90 (89.6) | 29 (3.2) |  |
| Yes | 93 (9.2) | 4 (4.3) | 0.16 |
| **Sore throat** |  |  |  |
| No | 841 (83.6) | 28 (3.3) |  |
| Yes | 160 (15.9) | 6 (3.8) | 0.81 |
| **Cough** |  |  |  |
| No | 759 (75.4) | 22 (2.9) |  |
| Yes | 244 (24.3) | 12 (4.9) | 0.15 |
| **Difficulty in breathing** |  |  |  |
| No | 725 (72.3) | 23 (3.2) |  |
| Yes | 278 (27.7) | 11 (4) | 0.56 |
| **Chills** |  |  |  |
| No | 905 (91.1) | 31 (3.5) |  |
| Yes | 88 (11.4) | 3 (2.6) | 1 |
| **Vomiting** |  |  |  |
| No | 885 (88.6) | 34 (3.5) |  |
| Yes | 114 (11.3) | 0 | 0.03 |
| **Nausea** |  |  |  |
| No | 976 (97.2) | 29 (3.2) |  |
| Yes | 28 (2.8) | 5 (5.2) | **0.002** |
| **Diarrhea** |  |  |  |
| No | 905 (90.3) | 33 (3.6) |  |
| Yes | 97 (9.7) | 0 | 0.07 |
| **Headache** |  |  |  |
| No | 600 (59.9) | 15 (2.5) |  |
| Yes | 401 (40.1) | 18 (4.5) | 0.10 |
| **Rash** |  |  |  |
| No | 972 (98.3) | 32 (3.3) |  |
| Yes | 16 (1.7) | 0 | 1 |
| **Conjunctivitis** |  |  |  |
| No | 943 (94.8) | 32 (3.4) |  |
| Yes | 52 (5.2) | 1 (1.9) | 1 |
| **Myalgia** |  |  |  |
| No | 700 (70.1) | 19 (2.7) |  |
| Yes | 299 (29.9) | 15 (5) | 0.09 |
| **Arthralgia** |  |  |  |
| No | 777 (77.8) | 10 (4.5) |  |
| Yes | 222 (22.2) | 24 (3.1) | **<0.001** |
| **Loss of Appetite** |  |  |  |
| No | 880 (87.6) | 27 (3.1) |  |
| Yes | 125 (12.4) | 7 (5.6) | 0.18 |
| **Anosmia** |  |  |  |
| No | 902 (89.7) | 30 (3.3) |  |
| Yes | 102 (10.1) | 4 (3.9) | 1 |
| **Fatigue** |  |  |  |
| No | 586 (58.4) | 12 (2) |  |
| Yes | 410 (41.7) | 22 (5.4) | 0.01 |
| **Seizure** |  |  |  |
| No | 487 (98.4) | 18 (3.7) |  |
| Yes | 8 (0.02) | 1 (12.5) | 0.27 |
| **Altered consciousness** |  |  |  |
| No | 985 (97.8) | 32 (3.2) |  |
| Yes | 11 (2.2 | 1 (9.1) | 0.31 |
